# Analyzing networks of phenotypes in complex diseases: methodology and applications in COPD

**DOI:** 10.1186/1752-0509-8-78

**Published:** 2014-06-25

**Authors:** Jen-hwa Chu, Craig P Hersh, Peter J Castaldi, Michael H Cho, Benjamin A Raby, Nan Laird, Russell Bowler, Stephen Rennard, Joseph Loscalzo, John Quackenbush, Edwin K Silverman

**Affiliations:** 1 Channing Division of Network Medicine, Brigham and Women’s Hospital, Boston, MA, USA; 2 Department of Medicine, Brigham and Women’s Hospital, Boston, MA, USA; 3 Division of Pulmonary and Critical Care Medicine, Brigham and Women’s Hospital, Boston, MA, USA; 4 Department of Biostatistics, Harvard School of Public Health, Boston, MA, USA; 5 Department of Medicine, National Jewish Health, Denver, CO, USA; 6 University of Nebraska Medical Center, Omaha, NE, USA; 7 Division of Cardiovascular Medicine, Brigham and Women’s Hospital, Boston, MA, USA; 8 Dana-Farber Cancer Institute, Boston, MA, USA

**Keywords:** Network medicine, Phenotypic networks, COPD, Genetic association analysis

## Abstract

**Background:**

The investigation of complex disease heterogeneity has been challenging. Here, we introduce a network-based approach, using partial correlations, that analyzes the relationships among multiple disease-related phenotypes.

**Results:**

We applied this method to two large, well-characterized studies of chronic obstructive pulmonary disease (COPD). We also examined the associations between these COPD phenotypic networks and other factors, including case-control status, disease severity, and genetic variants. Using these phenotypic networks, we have detected novel relationships between phenotypes that would not have been observed using traditional epidemiological approaches.

**Conclusion:**

Phenotypic network analysis of complex diseases could provide novel insights into disease susceptibility, disease severity, and genetic mechanisms.

## Background

Complex diseases like diabetes, stroke, many types of cancer, and chronic obstructive pulmonary disease (COPD) are likely heterogeneous syndromes composed of multiple disease subtypes that manifest a similar pathological or physiological outcome. These subtypes may have different genetic determinants. In order to understand this heterogeneity, a variety of clinical, physiological, imaging, pathological, and biochemical disease-related phenotypes have been analyzed [1]. In standard clinical epidemiological approaches, univariate and multivariate regression analyses are performed to determine significant and independent predictors of disease development. However, the available disease-related phenotypes may be crude assessments of disease pathophysiology; any analyses that are performed may be confounded by grouping multiple subtypes together. The challenge we face, in part, is deconvoluting these disease-related phenotypes and defining their relationships to one another and to specific genetic determinants.

Network analysis has the potential to provide a holistic approach to the understanding of disease complexity, rather than focusing on individual components of disease [2]. Network approaches can capture emergent properties that are not apparent when network components are analyzed in a pair-wise manner. However, network medicine approaches to complex diseases have largely focused on relating a disease to the underlying cellular and molecular interaction network [3]. Correlation-based networks have been frequently used to analyze gene expression data [4,5], but these methods have not been widely applied to the study of disease-related phenotypes. Barabási and colleagues [6] used diagnostic coding data to assess phenotypic network relationships between different disease categories, but not to analyze multiple quantitative phenotypes within one complex disease. Using COPD as an example, we describe the application of network inference methods to explore the relationships between disease-related phenotypes that have been found to be relevant in determining disease severity and outcome, and, ultimately, to begin to define the complex heterogeneity of the disease.

## Methods

### Network inference and comparison

To infer phenotypic networks, we used the Gaussian graphical model (GGM) introduced by [7] and [8]. Briefly, the model, which is based on the assumption that the variables have Gaussian distributions, infers the connection between each pair of variables and creates a phenotypic network based on partial correlations.

Assume that we have *P* phenotype variables and *K* subjects. We begin by constructing a *P*×*K* matrix, *Y*, where we assume that the elements of *Y* follow a multivariate normal distribution:

$$Y_{i}={\left(y_{1i},\mathrm{....}y_{\text{Pi}}\right)}^{T}∼N_{P}\left(\mu_{Y},\Sigma_{Y}\right),\;i=1,\mathrm{....K},$$

Here, *y*_*j* *i*_ represents the *j*th phenotype variable in the *i*th subject, *μ* is the mean vector and *Σ* is the covariance matrix. The covariance matrix *Σ*_*Y*_ and the partial correlation matrix (denoted by *Ω*) for *Y* are estimated (see [9]). The partial correlation (PCOR) *ω*_*j* *k*_ measures the correlation between variable *j* and variable *k* while controlling for all other variables. Therefore, *ω*_*j* *k*_ represents the conditional dependency between variable *j* and variable *k*, with *ω*_*j* *k*_=0 if the two variables are independent conditional on all other variables and *ω*_*j* *k*_≠0 if they are conditionally correlated. For each pair of variables that are conditionally dependent, the presumed causal relationship between the variables is a direct one and independent of all other variables. We assume that these partial correlations represent the hidden connections between phenotypic variables that may help to refine disease subtypes.

Under the null hypothesis in which all variables are independent, Hotelling [10] gives the null distribution of sample partial correlation *ω* as

$$p(\omega |\kappa )={\left(1-\omega^{2}\right)}^{\left(\kappa -3\right)/2}\frac{\Gamma (\kappa /2)}{\pi^{1/2}\Gamma \left[(\kappa -1)/2\right]},$$

where *κ* is the degrees of freedom (*K*−*P*+1). Therefore, we can compute the p-values for the estimated partial correlation coefficients for each pair of phenotypic variables and test for the presence of a significant connection between those variables in the phenotypic network. In addition, we can also test for differences in the network connectivity between two groups of subjects by permutation tests. For example, to test for differential connectivity between COPD cases and controls, we randomly swap the labels of cases and controls and calculate the PCORs in the shuffled groups, repeated 10,000 times, to obtain the distribution of PCORs under the null hypothesis in which the presence or absence of connections is not associated with the case-control status. The empirical p-values are reported. Analogously, we have also tested differential connectivity between different genotypes for two previously identified genome-wide significant SNPs associated with COPD using the same approach.

Opgen-Rhein and Strimmer [11] have extended the GGM method to infer the directionality of the edges between each pair of variables. They proposed a test of directionality based on the log-ratios of standardized partial variances. This method enables identification of a “partially directed graph” where some of the significant edges identified by GGM methods will have directions, which might imply causality, while other edges remain undirected.

### Study populations and phenotypic variable selection

COPD is a disease defined by abnormal physiology, with chronic airflow obstruction as the common, key feature [12]. Chronic airflow obstruction is characterized by reductions in the forced expiratory volume in one second (FEV1) and in the ratio of the FEV1 to the forced vital capacity (FVC), which are assessed by spirometry. Clinical epidemiological studies have identified multiple factors that contribute to COPD, including cigarette smoking (often quantified as pack-years, where an average of one pack of cigarettes smoked per day for one year is one pack-year) and increasing age. In addition, a variety of disease-related phenotypes have been studied related to imaging, exercise capacity, respiratory symptoms, and physiology. Computerized tomography (CT) imaging enables assessment of the severity and distribution of emphysema–the destruction of lung parenchyma–as well as thickening of airways [13-15]. The underlying assumption in our analysis is that these phenotypic variables are not independent, but, rather, interact to define distinct groups of patients (subtypes). By defining these subtypes, we might better be able to classify patients, understand their unique disease characteristics, and ultimately direct them to appropriate therapies.

The COPDGene Study [16] is a multi-center genetic and epidemiologic investigation to study COPD and other smoking-related lung diseases. In this study, 10,192 smokers (including 6,784 non-Hispanic Whites (NHW) and 3,408 African-Americans (AA)) have completed a detailed protocol, including questionnaires, pre-and post-bronchodilator spirometry, high-resolution CT scanning of the chest, exercise capacity (assessed by six minute walk distance), and blood samples for genotyping. Samples were genotyped using the Illumina OmniExpress platform, which assayed genetic polymorphisms at over 700,000 sites along the genome; the genotype data have gone through standard quality-control procedures for genome-wide association analysis. Briefly, a total of 221 subjects and 83,423 markers were excluded for quality control reasons, including identity-by-descent, gender mismatches, genotype missingness, Hardy-Weinberg disequilibrium in controls, and low minor allele frequency. The details of the quality control procedures are available at http://www.copdgene.org/sites/default/files/GWAS_QC_Methodology_20121115.pdf.

For phenotypic network analysis, we selected 10 key quantitative COPD-related phenotypes based on clinical experts’ opinions (co-authors EKS, CPH, and MHC). The phenotypes were chosen to represent major disease-related components, including imaging, physiology, exercise capacity, and exacerbations, as well as important demographic variables (Table 1). Although over 300 variables were captured by questionnaires, clinical assessments, and CT scanning in COPDGene, we chose phenotypes to avoid duplicate assessment of the same aspect of the disease (e.g., lung function, emphysema severity, and airway wall thickness). For example, we included FEV1 but excluded FEV1/FVC, as they are both lung function phenotypes which assess airflow obstruction. Subjects with missing data in any of the 10 quantitative variables were excluded. Therefore, a complete set of 8,141 subjects were used in the following analyses, including 5,478 NHWs and 2,514 AAs. Case subjects were defined by FEV1 <80*%* predicted and FEV1/FVC <0.7, while control subjects were defined by FEV1 ≥80*%* predicted and FEV1/FVC ≥0.7. In addition to assessment based on case-control status, we compared groups of subjects homozygous for risk- and non-risk alleles at known GWAS SNPs, excluding heterozygotes from the genotype-stratified phenotypic networks to maximize phenotypic effects. To assess the impact of including phenotypic variables that are not closely related to COPD on our phenotypic networks, we also created networks including heart rate and systolic blood pressure as well as networks including two randomly generated variables.

**Table 1:** Description of phenotypic variables

**Variables (abbreviation)**	**Descriptions/Comments**
FEV1 (% predicted FEV1)	Observed FEV1 (liters)/predicted FEV1 (liters), with predicted valued from Hankinson reference equations
Emphysema (Emph)	% Emphysema at -950 Hounsfield units(HU)
Emphysema Distribution (EmphDist)	Log ratio of emphysema at -950 HU in the upper 1/3 of lung fields compared to the lower 1/3 of lung fields
Gas Trapping (GasTrap)	Air trapping at -856HU on expiratory chest CT scan
Airway Wall Area (Pi10)	Square root of the wall area of a hypothetical 10 mm internal perimeter airway
Exacerbation frequency (ExacerFreq)	Number of COPD exacerbations during the year before study enrollment
Six minute walk distance (6MWD)	Measure of exercise capacity
BMI	Body Mass Index
Age	In years
Pack-Years (PackYear)	One pack-year is defined as smoking one pack (20 cigarettes) per day for one year

Evaluation of COPD Longitudinally to Identify Predictive Surrogate Endpoints (ECLIPSE, [17]) is a large longitudinal study of COPD patients and controls with comprehensive phenotyping similar to COPDGene. Therefore, we used a subset of 1,705 COPD cases (including 1,667 white subjects) with complete data for the 10 quantitative variables at their baseline study visit to build phenotypic networks. All variables in Table 1 were available in ECLIPSE, except for Emphysema Distribution and Gas Trapping. Therefore, networks with 8 variables were built for both COPDGene and ECLIPSE for comparison.

## Results

### Whole population phenotypic network in COPDGene

**Figure 1:**
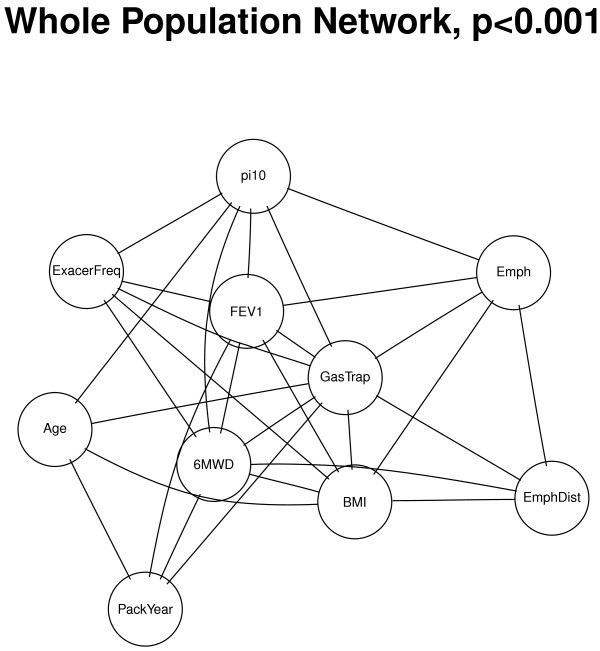
**Whole population network (N =8,141).** Undirected edges denote partial correlation coefficients that were significant at *p*<0.001.

The ten selected COPD-related phenotypes in COPDGene were found to be highly connected in the whole study population. Out of 45 pairs of phenotypes, 37 had significant PCORs with p-values <0.05, and 29 pairs were significant with p-values <0.001 (density = 64.44%, where the density of a network is defined by the portion of all possible connections in a network that are actual connections, see Figure 1 and Table 2). The most highly connected nodes were FEV1 and Gas Trapping (see Figure 1), with Gas Trapping significantly connected with all of the analyzed phenotypes. In addition, the 16 pairs that were not directly connected (p-values >0.001) were connected through only one transitive node based on shortest path analysis [18]. The majority of shortest paths connected through gas trapping (9 out of 16), suggesting that gas trapping is a “hub” in the phenotypic network. This finding is consistent with the high correlation observed between CT gas trapping and spirometric measures [19], and also with the observation that CT gas trapping encompasses the two major pathological processes in COPD–emphysema and small airway disease. Most edges in this whole population network remained statistically significant after we stratified by race, while the NHW network edges were slightly more significant than the AA network likely due to larger sample size and better power. FEV1 and Gas Trapping remained highly connected in the race-stratified networks. The top four pairs (CT Emphysema/Gas Trapping, FEV1/Gas Trapping, FEV1/Pi10, and Gas Trapping/Age) all stayed consistently top-ranked for the whole population and race-stratified networks and were all highly significant (see Table 2).

**Table 2:** Edges of whole population network with p-values < 0.001

**Node 1**	**Node 2**	**Whole population**		**NHW**		**AA**	
		**P-value**	**PCOR**	**P-value**	**PCOR**	**P-value**	**PCOR**
Emphysema	Gas Trapping	<E-238	0.654	<E-223	0.675	2.94E-239	0.594
FEV1% pred	Gas Trapping	1.88E-238	-0.353	5.59E-223	-0.411	5.64E-64	-0.328
FEV1% pred	Airway Wall Area	4.83E-193	-0.320	8.54E-138	-0.328	2.09E-38	-0.254
Gas Trapping	Age	2.04E-102	0.234	7.49E-92	0.269	1.36E-26	-0.210
Gas Trapping	BMI	7.47E-97	-0.228	1.07E-61	-0.221	2.13E-23	-0.197
6MWD	BMI	7.35E-73	-0.198	5.37E-56	-0.210	2.13E-23	0.197
Airway Wall Area	6MWD	5.29E-64	-0.185	4.66E-32	-0.158	4.78E-19	0.176
Age	Pack-years	7.47E-63	0.183	1.51E-23	0.134	1.75E-12	0.140
FEV1% pred	6MWD	6.49E-60	0.179	1.47E-76	0.246	4.05E-26	-0.208
FEV1% pred	Exacerbation Frequency	2.66E-37	-0.140	1.13E-23	-0.135	1.32E-08	-0.113
FEV1% pred	Emphysema	4.00E-37	-0.140	1.17E-09	-0.082	7.86E-06	-0.089
Emphysema	Airway Wall Area	9.27E-33	-0.131	1.16E-27	-0.146	2.85E-13	0.145
FEV1% pred	BMI	2.22E-25	-0.115	2.03E-17	-0.114	1.03E-06	-0.097
FEV1% pred	Pack-years	4.86E-24	-0.111	1.46E-20	-0.125	5.74E-06	0.090
6MWD	Pack-years	1.78E-16	-0.091	8.24E-23	-0.132	2.07E-07	-0.103
Gas Trapping	6MWD	7.75E-12	-0.075	0.2569	-0.015	0.3793	-0.017
Exacerbation Frequency	6MWD	2.07E-11	-0.074	9.03E-12	-0.092	0.00015	-0.075
Airway Wall Area	Age	7.17E-10	-0.068	0.3368	-0.012	0.5379	-0.012
Emphysema	Emphysema distribution	6.35E-07	0.0551	2.77E-05	0.056	0.005	0.055
Emphysema distribution	Gas Trapping	9.39E-07	-0.0543	0.0001	-0.051	4.55E-05	-0.081
BMI	Age	6.32E-06	0.0500	0.0061	0.037	0.1127	0.031
Gas Trapping	Exacerbation Frequency	9.07E-06	0.0491	0.0095	0.035	0.1506	-0.028
Emphysema distribution	BMI	1.19E-05	-0.0485	0.0493	-0.026	1.25E-05	-0.087
Emphysema	BMI	1.90E-05	-0.0473	5.06E-05	-0.054	4.55E-05	0.081
Gas Trapping	Airway Wall Area	2.86E-05	-0.0463	0.0121	-0.033	0.2662	0.022
Airway Wall Area	Exacerbation Frequency	5.50E-05	0.0447	8.66E-05	0.053	0.004	0.057
Gas Trapping	Pack-years	0.00011	0.0426	0.0002	0.049	0.027	0.044
Emphysema distribution	6MWD	0.00015	-0.0418	0.0006	-0.045	0.0015	-0.063
Exacerbation Frequency	BMI	0.00023	0.0407	0.1588	0.019	0.0908	-0.033

**Figure 2:**
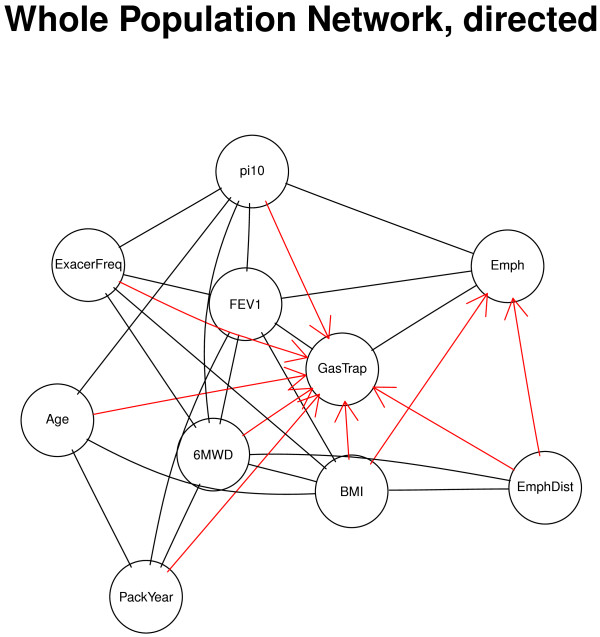
**Partially directed network from the whole COPDGene population (N =8,141).** The topology of the network is identical to the correlation graph in Figure 1, but the edges with significant directionality are oriented.

Since the ten variables were chosen based on their association with COPD, it was not surprising to find that most of the variables were highly connected. To assess the effects of variable selection, we repeated the analysis with two scenarios: (1) we added two extra variables randomly generated from a standard Gaussian distribution; and (2) we added two “extraneous” variables that were presumably less closely related to COPD: systolic blood pressure (SysBP) and heart rate (HR). As expected, the Gaussian random variables were not connected to any other variables. However, when the two “extraneous” clinical variables were introduced they were found to be connected to some of the other variables, but they were not an integral part of the graph. The network was sparser, as there were fewer edges between these presumably less related variables and other network components. Using the same threshold (*p*<0.001), 37 pairs of variables were significantly connected (density = 56.06%), including all 29 pairs that were significantly connected in the original network analysis. The extra 8 edges resulting from the two presumably unrelated variables included some clinically expected pairs including demographic variables, such as SysBP/BMI, SysBP/Age, and HR/Age. There were also a few connections between HR and COPD phenotypes which could be of potential interest (See Additional file 1: Figure S1). Therefore, variables selection does play an important role in the degree in which the variables are connected.Although our primary phenotypic network analysis was based on undirected edges, we also created a phenotypic network using directed edges–where they could be defined with certainty. The partially directed network analysis showed that for 9 out of the 29 edges directionality could be established, with 7 variables directed toward Gas Trapping (except for FEV1 and Emphysema, See Figure 2).

### Case-control phenotypic network comparison in COPDGene

**Figure 3:**
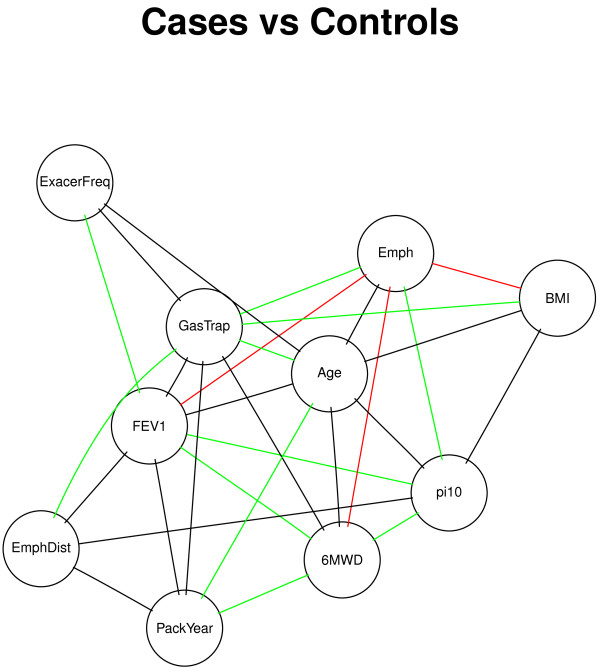
**Comparison of COPDGene case and control networks.** Undirected edges represent significantly different partial correlation coefficients between case and control subjects. The green edges are present in both groups (*p*<0.05) and the correlations are in the same direction of effect. The red edges are present in both groups but the correlations are in the opposite direction of effect. The black edges are present in one group but not the other.

We then built phenotypic networks for COPD case and control groups separately to examine the similarities and differences in phenotypic relationships between these two groups. Separate GGM networks were estimated in COPDGene for smoking controls with normal spirometry (*n*=3597) and COPD cases with moderate to very severe airflow obstruction (GOLD Stage ≥2,*n*=2894) to explore the impact of COPD on phenotypic relationships. Using p-value =0.05 as the threshold for statistical significance, the case and control networks each had 30 significant edges. The top pairs of variables were consistent in these two phenotypic networks, including CT Emphysema/Gas Trapping, Gas Trapping/BMI, Gas Trapping/Age, and CT Emphysema/Pi10, with a total of 17 edges present in both subgroups. However, the presence of these edges in both groups does not exclude the possibility that these partial correlations could be associated with case-control status. The permutation tests showed some additional differences between the networks, where 24 pairs with significantly different p-values in the comparison between case and control networks were observed (See Additional file 2: Table S1). For example, the Gas-Trapping/BMI pair had significant negative connections in both groups, but was more strongly connected in the case group than the control group. There were 32 pairs significantly associated with case-control status (See Figure 3). While most pairs had very similar patterns of correlation in both groups, one of the notable exceptions was between CT Emphysema and BMI. Higher CT emphysema was associated with higher BMI in the control group but was associated with lower BMI in the case group, and both associations were statistically significant.

### Moderate/Severe COPD network comparison in COPDGene

**Figure 4:**
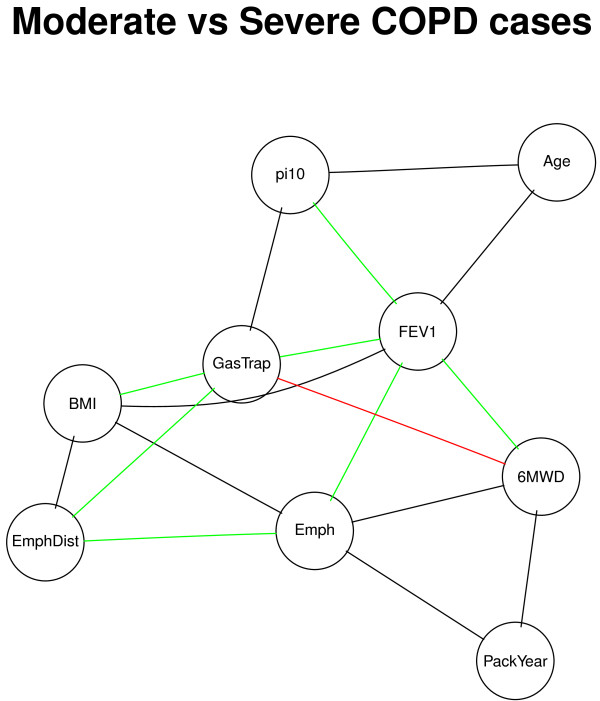
**Comparison of moderate and severe COPD networks.** Undirected edges represent significantly different partial correlation coefficients between moderate and severe COPD subjects. The green edges are present in both groups (*p*<0.05) and the correlations are in the same direction of effect. The red edges are present in both groups but the correlations are in the opposite direction of effect. The black edges are present in one group but not the other.

**Figure 5:**
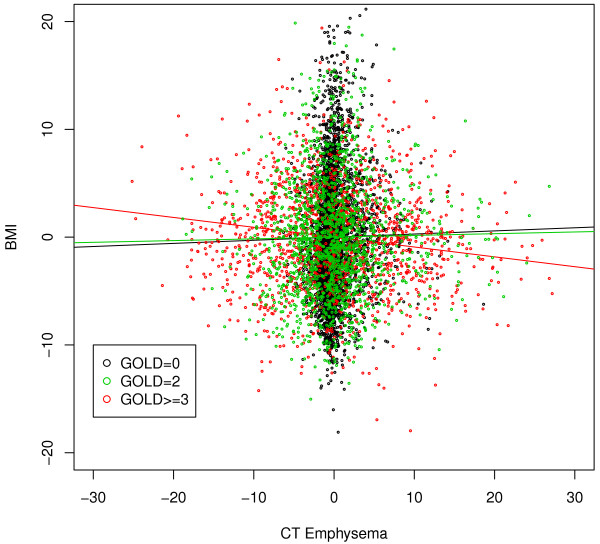
**Partial residual plot.** The partial residual plot between BMI and CT Emphysema for the smoking controls (black), moderate COPD cases (green), and severe COPD cases (red) networks. The partial residuals are the residuals of BMI and CT Emphysema from regressing out the other 8 variables.

Next, we constructed phenotypic networks in COPDGene moderate COPD cases (GOLD =2,*n*=1563) and severe to very severe COPD cases (GOLD ≥3,*n*=1331) to test for association between the phenotypic networks and disease severity. The moderate COPD network had 25 edges under the p-value <0.05 threshold, while the severe COPD network had 24–slightly fewer connections than the case-control networks in the section above, likely due to smaller sample size (See Additional file 2: Table S1). Globally, the differences between the two networks of COPD cases were less pronounced than the case-control network comparison, with only 17 pairs with significant differential connections according to the permutation testing (Figure 4). However, when we compared the smoking controls with the moderate and severe COPD case groups separately, we observed many pronounced differences in the control/severe COPD comparison, with multiple pairs significantly positively correlated in one network and negatively correlated in another. In many cases, we find the control group and severe COPD group at the opposite ends of the distribution, with the moderate COPD group in the middle. For example, CT Emphysema/6MWD had a negative correlation in the severe COPD network, no correlation in the moderate COPD group, and a strongly positive correlation in the control group. We also found that CT Emphysema and BMI were positively correlated in the control group, not correlated in the moderate COPD group, and negatively correlated in the severe COPD group. Figure 5 shows the BMI-CT Emphysema partial residual plot (the residuals of BMI and CT Emphysema from regressing out the other 8 variables) in the three groups, and we can see that the negative association between BMI and emphysema was only present in severe COPD cases. Table 3 shows the partial correlation coefficients and Pearson correlation coefficients between BMI and emphysema, and we observe that the opposite relationships between the control and COPD groups only became apparent after we regressed out the other 8 variables in the partial correlation framework. These results suggest that partial correlations could provide additional insight about the relationships between these phenotypes beyond standard epidemiological analyses.

**Table 3:** Partial correlation and Pearson correlation coefficients for BMI and CT emphysema

	**Control**	**Moderate COPD**	**Severe COPD**
Partial correlationcoefficients	0.07	0.008	-0.07
Pearson correlation coefficients	-0.07	-0.20	-0.46

### Genetic-based network comparison in COPDGene

We also constructed phenotypic networks for COPDGene subjects defined by their genotypes at two SNPs previously associated with COPD in genome-wide association studies: rs1980057 (*HHIP*) [20,21] and rs7671167 (*FAM13A*) [22]. Separate networks were built for homozygous samples (2 copies of the COPD-risk allele or 2 copies of the non-risk allele) for each of these SNPs. Note that in both loci, the minor allele has been associated with COPD protection. We only built genotype-stratified phenotypic networks for NHW subjects, as this *FAM13A* SNP did not have a significant association with COPD in the AA population in previous GWAS [23], and the *HHIP* SNP was a relatively uncommon variant in AA population (MAF =0.10) with few homozygous minor allele subjects.

**Figure 6:**
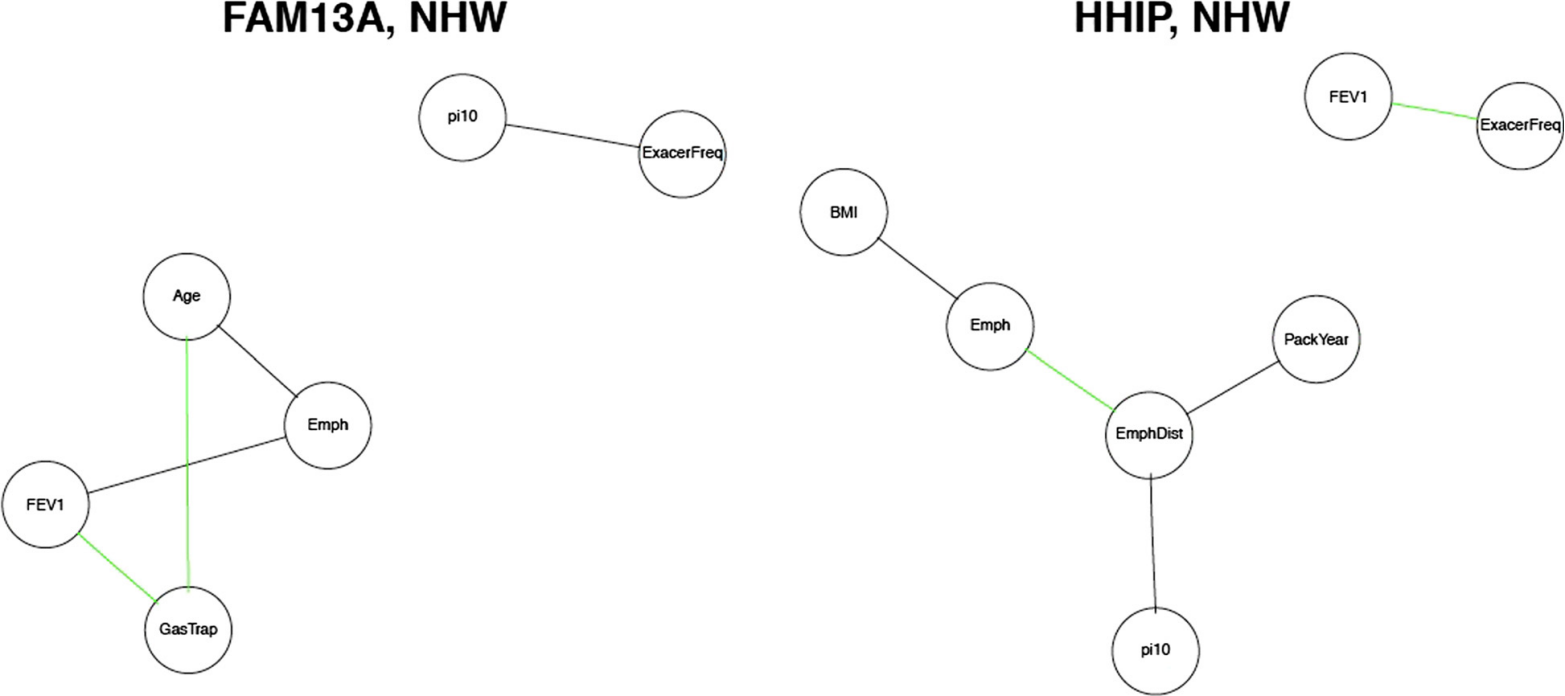
**Comparison of genetically perturbed networks.** (1) *HHIP* in non-Hispanic White (NHW) subjects (2 copies of the COPD-risk or non-risk allele) and (2) *FAM13A* NHW (2 copies of the COPD-risk or non-risk allele). The green edges are present in both groups (*p*<0.05) and the partial correlations have the same sign, but the magnitude of effect is significantly different between genotype groups. The black edges are present in one group but not the other.

Using permutation tests, we observed that only a few phenotype pairs significantly differed between these genotype-stratified phenotypic networks, and none of the edges was significant in opposite directions (See Figure 6 and Additional file 3: Table S2, Additional file 4: Table S3). The most discordant phenotype pair for *FAM13A* was FEV1/Emphysema, which was negatively correlated in the *FAM13A* COPD-non-risk group but not correlated in the *FAM13A* COPD-risk group. Other pairs that showed differential connection between the two groups include Pi10/Exacerbation Frequency and Age/CT Emphysema. For *HHIP*, we found FEV1/Exacerbation Frequency to be negatively correlated in both homozygous groups, but the partial correlation was significantly stronger in the COPD-non-risk group than in the COPD-risk group (−0.21 vs. −0.13). There were a few other pairs with only one homozygous group deviating from the null distribution (p-values <0.05) based on the permutation tests, and the signal was not as strong. Overall, the genetic variables did not have effects on the phenotypic networks that were as great as case-control status or COPD severity.

### ECLIPSE network comparison

Finally, we constructed phenotypic networks from ECLIPSE, another independent COPD population, and compared the results between the ECLIPSE and COPDGene networks. The major difference between these two cohorts is that ECLIPSE contains mostly moderate to severe COPD samples (GOLD 2-4) and mostly Caucasians. Therefore, we performed the comparative studies on two sets of sub-populations: (1) All COPD cases (GOLD 2-4, n =2,894 for COPDGene and n =1,705 for ECLIPSE); (2) NHW COPD cases only (n =2,264 for COPDGene and n =1,667 for ECLIPSE). Only 8 out of 10 variables in Table 1 were available in ECLIPSE, therefore the networks were built with 8 nodes and 28 possible edges. The results show that the networks from two populations were very similar (see Additional file 5: Table S4, Additional file 6: Table S5, and Additional file 7: Figure S2.), with minor differences. In all COPD cases, 15 pairs were significant with p <0.001 in COPDGene, out of which 12 were also significant in ECLIPSE (all of them were in the same direction). In white COPD cases, 16 pairs were significant with p <0.001 in COPDGene, out of which 13 were also significant in ECLIPSE. The most striking difference is that Pi10/BMI had the second highest correlation in ECLIPSE (in both analyses), but it was not significant in COPDGene. Overall, the networks from these two populations are reasonably comparable, and most of the strongest connections from COPDGene can be found in another independent population.

## Discussion

Complex diseases are assessed using an array of disease-related phenotypic variables, which may have subtle, hidden relationships that are not captured by standard epidemiological analyses. Understanding the relationships between these disease-related phenotypes in large, well-characterized study populations may provide insight into disease heterogeneity. Different approaches have been proposed to study the relationship between multiple phenotypes, including structural equation modeling [24] and mutual information [25]. We have developed an approach for constructing networks of phenotypic variables based on partial correlations between quantitative, disease-related phenotypes; for testing the statistical significance of those partial correlations within one phenotypic network; and for comparing those partial correlations between phenotypic networks constructed using different groups of subjects. The correlation-based networks that we analyzed are highly connected and not scale-free, as opposed to the sparse, scale-free networks that are observed in many biological and physical phenomena [2]. This is not surprising, as we built the networks based on a modest number of pre-selected variables closely related to the complex disease of interest.

The correlation-based networks have enabled us to detect novel relationships between disease-related phenotypes that would not have been observed in a single-variable analysis. Network based approaches are particularly useful in the studies of COPD, which is a complex disease with diverse clinical and molecular phenotypic profiles that might represent different subtypes [26]. In our study, the COPD network in the whole COPDGene study population provided a variety of clinically intuitive observations, such as the central location of gas trapping in the network–which includes both of the major COPD-related causes of airflow obstruction, emphysema and small airway disease. This key role of gas trapping was especially notable in the partially directed network (Figure 2). Comparisons between COPD cases and control subjects showed similar relationships for most variables, but an intriguing switch in the direction of the relationship between body mass index and CT emphysema was observed in controls compared to cases. Cachexia can accompany advanced COPD with severe emphysema, so a negative relationship between BMI and emphysema in COPD cases is clinically reasonable. Since the same radiation dose was used in all COPDGene subjects, the positive relationship between BMI and emphysema in control subjects could relate to the increased radiation noise with higher BMI, which could flatten the density histogram and artifactually increase the estimated degree of emphysema using densitometric thresholds.

Other phenotypic relationships are less intuitive but may point to important biological pathways. Comparison between moderate and severe/very severe COPD subjects showed a variety of interesting correlations between phenotypes. For example, increased emphysema was associated with reduced exercise capacity (6MWD) in severe COPD subjects but not in the moderate COPD group. Exercise capacity, assessed by 6MWD, includes many components but is likely significantly related to inspiratory capacity. In severe disease, inspiratory capacity is limited by baseline hyperinflation, which is observed by emphysema on CT scan. However, in moderate disease, other parameters are major determinants of 6MWD. Inspiratory capacity may be limiting with dynamic hyperinflation in moderate COPD subjects, but inspiratory capacity will likely not be closely associated with emphysema in this subgroup. It is unclear why airway wall area (Pi10) was significantly correlated with body mass index (BMI) in one of our study populations (ECLIPSE) but not the other (COPDGene). One possible explanation is that the CT radiation dose for ECLIPSE was substantially lower than in COPDGene, and this difference in radiation dose could have impacted how BMI influenced airway wall measurements.

Phenotypic networks have previously been studied in the context of multi-dimensional analyses that have included both phenotypic and genetic information [27,28]. Our method can also be applied in such integrative analyses. In particular, we examined the effects of genetic perturbations on the relationships between the phenotypes. In our COPD example, relatively few phenotypic interactions were different between homozygotes for alternative alleles of COPD GWAS regions near *HHIP* and *FAM13A*. Given the modest effects of these variants, and most other complex disease GWAS regions, these results are not surprising. However, the observed differences, such as the FEV1-emphysema relationship in alternate *FAM13A* genotypes, could provide clues regarding the underlying mechanisms by which these GWAS regions influence disease susceptibility. These results suggest that *FAM13A* may lead to reduced FEV1 through mechanisms other than increased emphysema, which is a testable hypothesis for future research. Similarly, the weaker relationship of FEV1 and exacerbation frequency in the COPD-associated group could indicate that any relationship of the *HHIP* locus to exacerbations may not be mediated through reduced FEV1.

Although published studies have described methods for assessing relationships between disease diagnostic categories in a network context [6,29], we instead focused on multiple disease-related phenotypes within one complex disease. While we believe this represents an important new approach, several limitations of our work need to be acknowledged. It is not clear whether it is preferable to use a weighted network, in which all edges are present but of variable magnitude, or an unweighted network, with an admittedly somewhat arbitrary threshold for placing an edge. Further work will also be required to determine the optimal approach for assessing the impact of genetic factors on phenotypic networks. We have compared alternate homozygous classes, but that approach eliminates the information in the typically larger heterozygous genotype group.

## Conclusion

In conclusion, we have presented a framework for analyzing and comparing partial correlations between multiple, quantitative disease-related phenotypes in networks. These phenotypic networks could provide insights into disease susceptibility, disease severity, and genetic mechanisms. Future directions will involve refining the approaches for selecting phenotypes to include in such networks as well as improved approaches for incorporating genetic information. Ultimately, these phenotypic networks may prove useful in developing novel classification systems for complex diseases.

## Competing interests

We have the following competing interests to report:

Edwin K. Silverman: Honoraria and consulting fees from Merck; grant support and consulting fees from GlaxoSmithKline; and consulting fees from Astra Zeneca.

Joseph Loscalzo: Ownership of shares in DZZOM, a start-up company.

Craig Hersh: Speaking fee from Novartis. Consultant for CSL Behring.

Michael Cho: Consulting fees from Merck.

## Authors’ contributions

JC developed the main mathematical models and implemented the algorithm. Additional analyses and interpretations of the results were performed by CPH, PJC, MHC, BAR, NL, RB, SR, JL and JQ. EKS is principal investigator of the primary grant supporting this work, “Genetics Epidemiology of COPD” and together with JC conceptualized the algorithm. JC and EKS were responsible for manuscript preparation. All authors have read the manuscript and approved the final version.

## Supplementary Material

Additional file 1 **Figure S1.** Whole population network (N =8,141) with two “extraneous” variables(yellow). Edges denote partial correlation coefficients that were significant at *p*<0.001. Click here for file

Additional file 2 **Table S1.** Raw p-values, partial correlations and permutation-based p-values for all edges for different COPD status groups. Click here for file

Additional file 3 **Table S2.** Raw p-values, partial correlations and permutation-based p-values for non-Hispanic White (NHW) populations with 2 copies of COPD risk or COPD non-risk allele (HHIP). Click here for file

Additional file 4 **Table S3.** Raw p-values, partial correlations and permutation-based p-values for non-Hispanic White (NHW) populations with 2 copies of COPD risk or COPD non-risk allele (FAM13A). Click here for file

Additional file 5 **Table S4.** Raw p-values and partial correlations for all edges in COPDGene and ECLIPSE for all cases. Click here for file

Additional file 6 **Table S5.** Raw p-values and partial correlations for all edges in COPDGene and ECLIPSE for all white cases. Click here for file

Additional file 7 **Figure S2.** Comparison of ECLIPSE and COPDGene networks on all cases and white cases only. Undirected edges denote partial correlation coefficients that were significant at *p*<0.001. Click here for file
