# Supplementary figures and images for: Analyzing networks of phenotypes in complex diseases: methodology and applications in COPD
Source: BMC Syst Biol. 2014 Jun 25;8:78. doi: 10.1186/1752-0509-8-78 (PMC4105829; doi:10.1186/1752-0509-8-78)

# Whole Population Network, $p < 0.001$

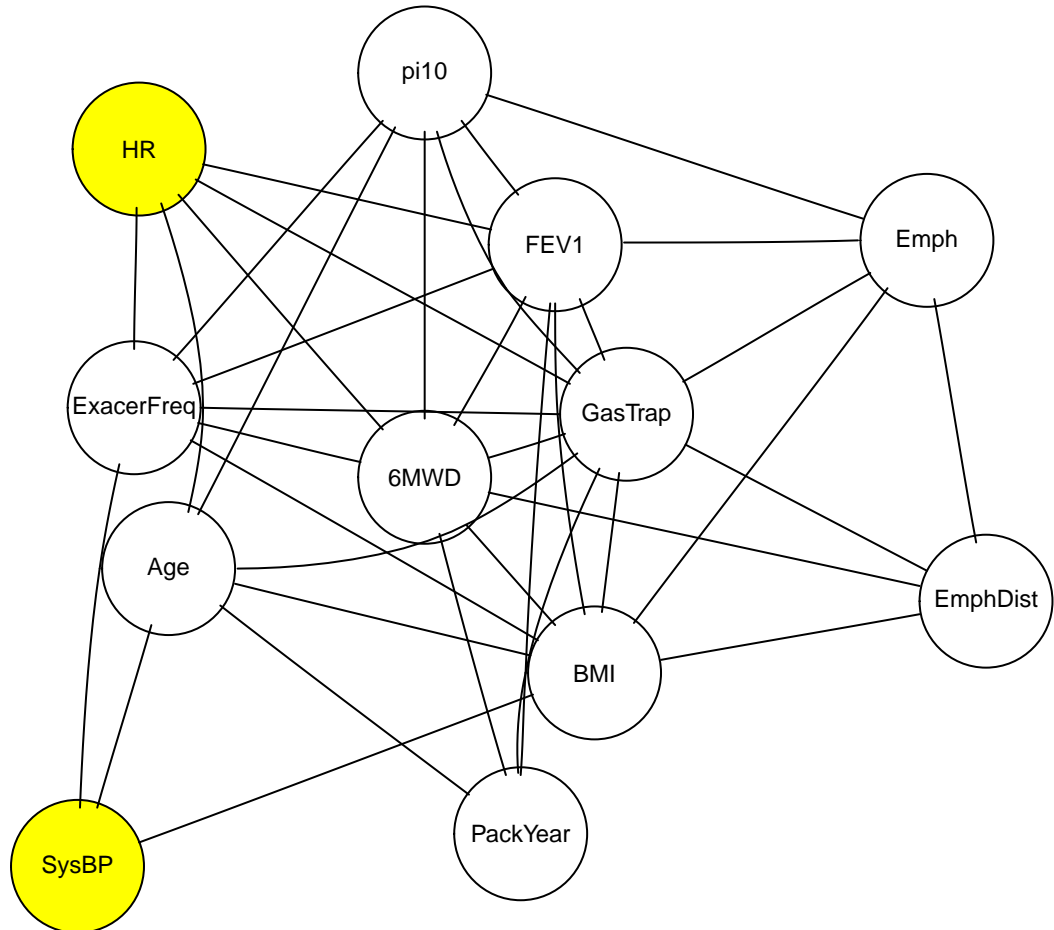

Supplement: Additional file 1 — Figure S1. Whole population network (N =8,141) with two “extraneous” variables(yellow). Edges denote partial correlation coefficients that were significant at p<0.001. [file 1752-0509-8-78-S1.pdf]
