# Supplementary material for: Analyzing networks of phenotypes in complex diseases: methodology and applications in COPD
Source: BMC Syst Biol. 2014 Jun 25;8:78. doi: 10.1186/1752-0509-8-78 (PMC4105829; doi:10.1186/1752-0509-8-78)
Supplement: Additional file 2 — Table S1. Raw p-values, partial correlations and permutation-based p-values for all edges for different COPD status groups. [file 1752-0509-8-78-S2.pdf]

	Node 1	Node 2	GOLD=0 P-value (Control)	GOLD>=2 p-value (Case)	Gold=2 p-value (moderate)	GOLD>=3 p-value (Severe)
1	Emphysema	Gas Trapping	1.07E-175	0.00E+00	8.89E-184	3.45E-150
2	Gas Trapping	BMI	3.34E-30	4.80E-48	2.02E-23	1.06E-31
3	Gas Trapping	Age	3.81E-28	8.86E-36	1.26E-16	6.44E-16
4	Emphysema	Airway Wall Area	8.66E-27	5.53E-23	1.14E-11	2.45E-10
5	6MWD	BMI	6.15E-26	9.97E-24	2.86E-16	1.37E-09
6	Airway Wall Area	6MWD	2.72E-23	3.87E-12	6.46E-06	2.85E-06
7	Age	Pack-years	3.18E-19	5.11E-22	5.08E-12	2.73E-10
8	Gas Trapping	6MWD	2.97E-18	0.8002	0.0042	0.0032
9	Emphysema	6MWD	2.10E-17	0.0029	0.1724	0.0001
10	6MWD	Pack-years	1.46E-15	0.0004	9.48E-06	0.6282
11	FEV1% pred	BMI	5.43E-10	6.23E-08	3.27E-05	0.7150
12	FEV1% pred	Airway Wall Area	1.07E-08	1.92E-58	1.92E-14	5.09E-14
13	Emphysema	Age	4.74E-07	0.1503	0.6508	0.4983
14	Exacerbation Frequency	6MWD	4.65E-06	2.46E-06	1.34E-06	0.0451
15	Emphysema	Emphysema Distribution	6.34E-06	1.60E-05	1.18E-12	0.0315
16	FEV1% pred	Emphysema	9.35E-06	8.01E-08	0.0069	0.0051
17	Emphysema	BMI	1.48E-05	0.0001	0.7461	0.0140
18	Emphysema Distribution	BMI	1.64E-05	0.5983	4.51E-05	0.1855
19	Emphysema Distribution	Airway Wall Area	1.97E-05	0.8263	0.3670	0.8338
20	Emphysema Distribution	Pack-years	0.0002	0.9547	0.9462	0.6874
21	Airway Wall Area	BMI	0.0003	0.5511	0.5282	0.6508
22	FEV1% pred	Pack-years	0.0005	0.4973	0.5306	0.0635
23	Exacerbation Frequency	BMI	0.0008	0.0687	0.0889	0.5097
24	FEV1% pred	Exacerbation Frequency	0.0018	1.81E-06	0.0041	0.0046
25	Gas Trapping	Airway Wall Area	0.0063	0.0406	0.8633	0.0033
26	Airway Wall Area	Exacerbation Frequency	0.0091	0.0123	0.0931	0.0445
27	FEV1% pred	6MWD	0.0107	3.73E-45	4.64E-07	5.46E-25
28	Emphysema Distribution	6MWD	0.0160	0.0134	0.0182	0.1041
29	Emphysema Distribution	Gas Trapping	0.0347	2.07E-05	1.55E-08	0.0329
30	Emphysema Distribution	Age	0.0424	0.0284	0.0174	0.1697
31	Airway Wall Area	Age	0.0516	0.0285	0.0001	0.2416
32	FEV1% pred	Emphysema Distribution	0.0822	0.7740	0.7391	0.7008
33	Airway Wall Area	Pack-years	0.0890	0.8360	0.7846	0.9626
34	6MWD	Age	0.1041	8.28E-07	0.0037	8.24E-06
35	BMI	Age	0.1110	2.12E-06	0.0007	0.0643
36	FEV1% pred	Gas Trapping	0.2125	4.48E-133	6.79E-15	6.53E-34
37	Exacerbation Frequency	Age	0.2707	1.90E-05	0.0190	0.0003
38	Emphysema	Pack-years	0.2911	0.0238	0.0542	0.0076
39	Emphysema	Exacerbation Frequency	0.3110	0.7687	0.2180	0.5722
40	Gas Trapping	Exacerbation Frequency	0.5458	0.0773	0.2741	0.4850
41	Exacerbation Frequency	Pack-years	0.6904	0.3498	0.6985	0.2569
42	Emphysema Distribution	Exacerbation Frequency	0.7295	0.8128	0.7598	0.9829
43	FEV1% pred	Age	0.7375	2.62E-10	0.6621	2.36E-10
44	BMI	Pack-years	0.8928	0.0722	0.7600	0.0799
45	Gas Trapping	Pack-years	0.9744	0.0045	0.2041	0.2517

Table S1: p-values for all edges for different COPD status groups

	Node 1	Node 2	GOLD=0 PCOR (Control)	GOLD>=2 PCOR (Case)	GOLD=2 PCOR (moderate)	GOLD>=3 PCOR (severe)
1	Emphysema	Gas Trapping	0.45	0.64	0.65	0.63
2	Gas Trapping	BMI	-0.19	-0.27	-0.25	-0.31
3	Gas Trapping	Age	0.18	0.23	0.21	0.22
4	Emphysema	Airway Wall Area	-0.18	-0.18	-0.17	-0.17
5	6MWD	BMI	-0.17	-0.19	-0.21	-0.17
6	Airway Wall Area	6MWD	-0.16	-0.13	-0.11	-0.13
7	Age	Pack-years	0.15	0.18	0.17	0.17
8	Gas Trapping	6MWD	-0.14	0.00	-0.07	0.08
9	Emphysema	6MWD	0.14	-0.06	0.03	-0.11
10	6MWD	Pack-years	-0.13	-0.07	-0.11	-0.01
11	FEV1% pred	BMI	-0.10	-0.10	-0.11	0.01
12	FEV1% pred	Airway Wall Area	-0.10	-0.29	-0.19	-0.21
13	Emphysema	Age	0.08	-0.03	0.01	-0.02
14	Exacerbation Frequency	6MWD	-0.08	-0.09	-0.12	-0.06
15	Emphysema	Emphysema Distribution	0.08	0.08	0.18	0.06
16	FEV1% pred	Emphysema	0.07	-0.10	-0.07	-0.08
17	Emphysema	BMI	0.07	-0.07	0.01	-0.07
18	Emphysema Distribution	BMI	-0.07	-0.01	-0.10	0.04
19	Emphysema Distribution	Airway Wall Area	0.07	0.00	0.02	-0.01
20	Emphysema Distribution	Pack-years	0.06	0.00	0.00	-0.01
21	Airway Wall Area	BMI	0.06	0.01	0.02	0.01
22	FEV1% pred	Pack-years	-0.06	-0.01	0.02	-0.05
23	Exacerbation Frequency	BMI	0.06	0.03	0.04	0.02
24	FEV1% pred	Exacerbation Frequency	-0.05	-0.09	-0.07	-0.08
25	Gas Trapping	Airway Wall Area	-0.05	-0.04	0.00	-0.08
26	Airway Wall Area	Exacerbation Frequency	0.04	0.05	0.04	0.06
27	FEV1% pred	6MWD	0.04	0.26	0.13	0.28
28	Emphysema Distribution	6MWD	-0.04	-0.05	-0.06	-0.04
29	Emphysema Distribution	Gas Trapping	-0.04	-0.08	-0.14	-0.06
30	Emphysema Distribution	Age	-0.03	-0.04	-0.06	-0.04
31	Airway Wall Area	Age	-0.03	-0.04	-0.10	0.03
32	FEV1% pred	Emphysema Distribution	-0.03	0.01	0.01	0.01
33	Airway Wall Area	Pack-years	-0.03	0.00	-0.01	0.00
34	6MWD	Age	0.03	-0.09	-0.07	-0.12
35	BMI	Age	0.03	0.09	0.09	0.05
36	FEV1% pred	Gas Trapping	-0.02	-0.43	-0.20	-0.32
37	Exacerbation Frequency	Age	0.02	-0.08	-0.06	-0.10
38	Emphysema	Pack-years	0.02	-0.04	0.05	-0.07
39	Emphysema	Exacerbation Frequency	0.02	-0.01	0.03	-0.02
40	Gas Trapping	Exacerbation Frequency	0.01	0.03	0.03	0.02
41	Exacerbation Frequency	Pack-years	-0.01	-0.02	-0.01	-0.03
42	Emphysema Distribution	Exacerbation Frequency	-0.01	0.00	0.01	0.00
43	FEV1% pred	Age	-0.01	0.12	-0.01	0.17
44	BMI	Pack-years	0.00	0.03	0.01	0.05
45	Gas Trapping	Pack-years	0.00	0.05	0.03	0.03

Table S1 (cont.): partial correlations for all edges for different COPD status groups

	Node 1	Node 2	Perm Pval GOLD=0 (control)	Perm Pval GOLD>=2 (case)	Perm Pval GOLD=2 (moderate)	Perm Pval GOLD>=3 (severe)
1	Emphysema	Gas Trapping	0	0.9808	0.3518	0.8558
2	Gas Trapping	BMI	0	0.0066	0.531	0.0024
3	Gas Trapping	Age	0	0.7922	0.2956	0.9624
4	Emphysema	Airway Wall Area	0.0024	0.0036	0.531	0.7482
5	6MWD	BMI	0.6018	0.7232	0.4316	0.2412
6	Airway Wall Area	6MWD	0.5244	0.0012	0.4882	0.9066
7	Age	Pack-years	0.0062	0.978	0.7298	0.753
8	Gas Trapping	6MWD	0	0	0.0072	0.017
9	Emphysema	6MWD	0.003	0.0144	0.7594	0.0042
10	6MWD	Pack-years	0.0006	0.0628	0.058	0.0056
11	FEV1% pred	BMI	0.4118	0.4066	0.342	8.00E-04
12	FEV1% pred	Airway Wall Area	0	0.9204	0	2.00E-04
13	Emphysema	Age	0.0006	0.302	0.5702	0.9808
14	Exacerbation Frequency	6MWD	0.8302	0.5492	0.1392	0.0646
15	Emphysema	Emphysema Distribution	0.3376	0.4012	0	0.0274
16	FEV1% pred	Emphysema	4.00E-04	0.1792	0	0.0044
17	Emphysema	BMI	0.0066	0.0504	0.0016	0.5482
18	Emphysema Distribution	BMI	0.3404	0.2012	0.035	0.6342
19	Emphysema Distribution	Airway Wall Area	0.001	0.1576	0.5056	0.4982
20	Emphysema Distribution	Pack-years	0.0068	0.0322	0.2686	0.9346
21	Airway Wall Area	BMI	0.0054	0.2246	0.943	0.6908
22	FEV1% pred	Pack-years	0	0	0.7616	0.0814
23	Exacerbation Frequency	BMI	0.1084	0.8046	0.7726	0.3548
24	FEV1% pred	Exacerbation Frequency	0	0	0.4448	0.7154
25	Gas Trapping	Airway Wall Area	0.1062	0.4174	0.1552	0.0236
26	Airway Wall Area	Exacerbation Frequency	0.5798	0.813	0.9082	0.6548
27	FEV1% pred	6MWD	0	0	0	0.054
28	Emphysema Distribution	6MWD	0.9604	0.883	0.5006	0.5982
29	Emphysema Distribution	Gas Trapping	0	0.4616	0.092	0.0376
30	Emphysema Distribution	Age	0.29	0.1616	0.444	0.513
31	Airway Wall Area	Age	0.0348	0.2766	0.025	0.2118
32	FEV1% pred	Emphysema Distribution	0.6946	0.0054	0.6958	0.4886
33	Airway Wall Area	Pack-years	0.3906	0.1888	0.6044	0.0746
34	6MWD	Age	0.1178	0.0092	0.4922	0.0784
35	BMI	Age	0.001	0.0434	0.78	0.0986
36	FEV1% pred	Gas Trapping	0	0.281	0	0.0124
37	Exacerbation Frequency	Age	0.1034	0.004	0.439	0.2154
38	Emphysema	Pack-years	0.0684	0.8348	0.0966	0.0244
39	Emphysema	Exacerbation Frequency	0.5656	0.436	0.1668	0.9252
40	Gas Trapping	Exacerbation Frequency	0.0066	0.5338	0.6762	0.4782
41	Exacerbation Frequency	Pack-years	0.406	0.9026	0.6628	0.5014
42	Emphysema Distribution	Exacerbation Frequency	0.8294	0.7476	0.8898	0.0506
43	FEV1% pred	Age	0.0956	0.0026	0	0.0012
44	BMI	Pack-years	0.0798	0.3096	0.1924	0.3502
45	Gas Trapping	Pack-years	2.00E-04	0.4822	0.1962	0.345

Table S1 (cont.): permutation-based p-values for all edges for different COPD status groups
