# Supplementary material for: Analyzing networks of phenotypes in complex diseases: methodology and applications in COPD
Source: BMC Syst Biol. 2014 Jun 25;8:78. doi: 10.1186/1752-0509-8-78 (PMC4105829; doi:10.1186/1752-0509-8-78)
Supplement: Additional file 3 — Table S2. Raw p-values, partial correlations and permutation-based p-values for non-Hispanic White (NHW) populations with 2 copies of COPD risk or COPD non-risk allele (HHIP). [file 1752-0509-8-78-S3.pdf]

	Node 1	Node 2	Population P-value	2 copies COPD risk allele p-value	2 copies COPD non-risk allele p-value
1	Emphysema	Gas Trapping	0	2.26E-239	8.67E-107
2	FEV1% pred	Gas Trapping	1.88E-238	8.39E-82	3.99E-31
3	FEV1% pred	Airway Wall Area	4.83E-193	8.30E-51	5.16E-24
4	Gas Trapping	Age	2.04E-102	1.63E-30	2.32E-18
5	FEV1% pred	6MWD	7.47E-97	1.45E-25	8.20E-14
6	Gas Trapping	BMI	7.35E-73	6.29E-24	2.00E-10
7	6MWD	BMI	5.29E-64	3.88E-23	1.57E-11
8	Airway Wall Area	6MWD	7.47E-63	8.20E-16	0.0001
9	Emphysema	Airway Wall Area	6.49E-60	2.68E-11	0.0001
10	FEV1% pred	Exacerbation Frequency	2.66E-37	2.43E-09	3.99E-10
11	Age	Pack-years	4.00E-37	2.79E-11	0.0003
12	6MWD	Pack-years	9.27E-33	1.01E-08	0.0135
13	6MWD	Age	2.22E-25	1.32E-08	4.70E-06
14	FEV1% pred	Pack-years	4.86E-24	4.71E-07	1.30E-07
15	FEV1% pred	BMI	1.78E-16	5.82E-08	0.0399
16	FEV1% pred	Age	7.75E-12	0.0001	0.0040
17	Exacerbation Frequency	6MWD	2.07E-11	0.0003	0.0916
18	Emphysema	Age	7.17E-10	0.0185	0.0110
19	FEV1% pred	Emphysema	6.35E-07	6.35E-06	0.0009
20	Exacerbation Frequency	Age	9.39E-07	0.0077	0.0079
21	Emphysema	Emphysema Distribution	6.32E-06	0.0072	0.0003
22	Emphysema	BMI	9.07E-06	0.0002	0.6357
23	Airway Wall Area	Exacerbation Frequency	1.19E-05	0.0046	0.6228
24	Emphysema Distribution	Gas Trapping	1.90E-05	0.0003	0.0378
25	Gas Trapping	Pack-years	2.86E-05	0.0134	0.6678
26	Emphysema Distribution	6MWD	5.50E-05	0.0006	0.4296
27	Emphysema	Pack-years	0.0001	0.0388	0.9147
28	BMI	Age	0.0002	0.0536	0.6084
29	Emphysema	6MWD	0.0002	0.0041	0.8322
30	Gas Trapping	Exacerbation Frequency	0.0015	0.0945	0.3691
31	Gas Trapping	Airway Wall Area	0.0015	0.1047	0.2591
32	Emphysema Distribution	Age	0.0035	0.0990	0.0654
33	Airway Wall Area	Pack-years	0.0046	0.3068	0.0595
34	Emphysema Distribution	BMI	0.0068	0.0001	0.0042
35	FEV1% pred	Emphysema Distribution	0.0166	0.0133	0.7722
36	BMI	Pack-years	0.0252	0.2597	0.1214
37	Airway Wall Area	BMI	0.0315	0.1670	0.4113
38	Exacerbation Frequency	BMI	0.0967	0.0508	0.5064
39	Exacerbation Frequency	Pack-years	0.1550	0.3038	0.1009
40	Gas Trapping	6MWD	0.2014	0.7065	0.1881
41	Airway Wall Area	Age	0.2467	0.7821	0.8489
42	Emphysema Distribution	Exacerbation Frequency	0.3621	0.8236	0.2072
43	Emphysema Distribution	Airway Wall Area	0.4888	0.9904	0.5390
44	Emphysema	Exacerbation Frequency	0.7714	0.8116	0.0643
45	Emphysema Distribution	Pack-years	0.8382	0.7588	0.0037

Table S2: p-values for all edges for non-Hispanic White (NHW) populations with 2 copies of COPD risk or COPD non-risk allele (HHIP)

	Node 1	Node 2	Population PCOR	2 copies COPD risk allele PCOR	2 copies COPD non-risk allele PCOR	2 copies COPD risk allele perm p-value	2 copies COPD non-risk allele perm p-value
1	Emphysema	Gas Trapping	0.6541	0.6509	0.6531	0.5842	0.6892
2	FEV1% pred	Gas Trapping	-0.3538	-0.4113	-0.3793	0.5802	0.4366
3	FEV1% pred	Airway Wall Area	-0.3200	-0.3275	-0.3335	0.8898	0.696
4	Gas Trapping	Age	0.2350	0.2538	0.2905	0.1864	0.2654
5	FEV1% pred	6MWD	-0.2285	0.2316	0.2496	0.613	0.5914
6	Gas Trapping	BMI	-0.1982	-0.2238	-0.2136	0.7808	0.8606
7	6MWD	BMI	-0.1857	-0.2199	-0.2261	0.866	0.847
8	Airway Wall Area	6MWD	0.1840	-0.1794	-0.1328	0.2424	0.2424
9	Emphysema	Airway Wall Area	0.1796	-0.1489	-0.1298	0.5874	0.656
10	FEV1% pred	Exacerbation Frequency	-0.1408	-0.1335	-0.2101	0.0278	0.041
11	Age	Pack-years	-0.1405	0.1488	0.1233	0.3526	0.5706
12	6MWD	Pack-years	-0.1316	-0.1282	-0.0838	0.2886	0.278
13	6MWD	Age	-0.1151	-0.1272	-0.1546	0.4474	0.4584
14	FEV1% pred	Pack-years	-0.1118	-0.1128	-0.1779	0.0652	0.0996
15	FEV1% pred	BMI	-0.0911	-0.1214	-0.0697	0.2426	0.2466
16	FEV1% pred	Age	-0.0758	0.0885	0.0974	0.5864	0.7912
17	Exacerbation Frequency	6MWD	-0.0742	-0.0813	-0.0573	0.7072	0.547
18	Emphysema	Age	-0.0683	-0.0529	-0.0862	0.1892	0.3604
19	FEV1% pred	Emphysema	0.0552	-0.1012	-0.1121	0.958	0.8986
20	Exacerbation Frequency	Age	-0.0543	-0.0598	-0.0901	0.3554	0.459
21	Emphysema	Emphysema Distribution	0.0500	0.0603	0.1229	0.3166	0.0186
22	Emphysema	BMI	0.0492	-0.0837	-0.0161	0.0546	0.0294
23	Airway Wall Area	Exacerbation Frequency	-0.0485	0.0636	0.0167	0.2218	0.2514
24	Emphysema Distribution	Gas Trapping	-0.0474	-0.0815	-0.0705	0.6964	0.829
25	Gas Trapping	Pack-years	-0.0464	0.0555	0.0146	0.276	0.2576
26	Emphysema Distribution	6MWD	0.0447	-0.0767	-0.0268	0.4116	0.0736
27	Emphysema	Pack-years	0.0427	-0.0464	0.0036	0.2432	0.1262
28	BMI	Age	-0.0419	0.0433	0.0174	0.6022	0.4714
29	Emphysema	6MWD	0.0408	-0.0644	0.0072	0.1142	0.1188
30	Gas Trapping	Exacerbation Frequency	-0.0353	0.0376	0.0305	0.7038	0.8814
31	Gas Trapping	Airway Wall Area	-0.0353	-0.0364	-0.0383	0.8808	0.9468
32	Emphysema Distribution	Age	0.0324	-0.0371	-0.0625	0.6276	0.5324
33	Airway Wall Area	Pack-years	-0.0314	-0.0230	-0.0640	0.297	0.3234
34	Emphysema Distribution	BMI	-0.0300	-0.0873	-0.0969	0.769	0.5812
35	FEV1% pred	Emphysema Distribution	0.0266	-0.0556	-0.0098	0.5314	0.1176
36	BMI	Pack-years	0.0248	0.0253	0.0526	0.6306	0.4378
37	Airway Wall Area	BMI	-0.0238	-0.0310	0.0279	0.1736	0.7306
38	Exacerbation Frequency	BMI	-0.0184	0.0439	-0.0226	0.081	0.9112
39	Exacerbation Frequency	Pack-years	-0.0158	-0.0231	0.0557	0.1422	0.1054
40	Gas Trapping	6MWD	0.0142	-0.0085	-0.0447	0.3652	0.3864
41	Airway Wall Area	Age	0.0128	-0.0062	-0.0065	0.6826	0.3418
42	Emphysema Distribution	Exacerbation Frequency	-0.0101	-0.0050	0.0428	0.681	0.1796
43	Emphysema Distribution	Airway Wall Area	-0.0077	0.0003	0.0209	0.0236	0.8622
44	Emphysema	Exacerbation Frequency	0.0032	0.0054	-0.0628	0.4022	0.1948
45	Emphysema Distribution	Pack-years	-0.0023	0.0069	0.0983	0.1542	0.0184

Table S2 (cont.): partial correlations and permutation-based p-values for all edges for non-Hispanic White (NHW) populations with 2 copies of COPD risk or COPD non-risk allele (HHIP)
