# Supplementary material for: Analyzing networks of phenotypes in complex diseases: methodology and applications in COPD
Source: BMC Syst Biol. 2014 Jun 25;8:78. doi: 10.1186/1752-0509-8-78 (PMC4105829; doi:10.1186/1752-0509-8-78)
Supplement: Additional file 4 — Table S3. Raw p-values, partial correlations and permutation-based p-values for non-Hispanic White (NHW) populations with 2 copies of COPD risk or COPD non-risk allele (FAM13A). [file 1752-0509-8-78-S4.pdf]

	Node 1	Node 2	Population P-value	2 copies COPD risk allele p-value	2 copies COPD non-risk allele p-value
1	Emphysema	Gas Trapping	0	2.75E-194	3.27E-165
2	FEV1 %pred	Gas Trapping	1.88E-238	1.51E-68	3.55E-43
3	FEV1 %pred	Airway Wall Area	4.83E-193	1.43E-36	8.49E-29
4	Gas Trapping	Age	2.04E-102	1.98E-32	2.15E-16
5	FEV1 %pred	6MWD	7.47E-97	3.58E-19	1.26E-18
6	Gas Trapping	BMI	7.35E-73	4.62E-15	6.36E-11
7	6MWD	BMI	5.29E-64	2.62E-18	4.54E-12
8	Airway Wall Area	6MWD	7.47E-63	1.96E-08	1.00E-08
9	Emphysema	Airway Wall Area	6.49E-60	1.34E-06	2.15E-08
10	FEV1 %pred	Exacerbation Frequency	2.66E-37	3.78E-08	3.10E-07
11	Age	Pack-years	4.00E-37	0.0002	5.79E-07
12	6MWD	Pack-years	9.27E-33	2.26E-09	0.0002
13	6MWD	Age	2.22E-25	1.01E-06	3.26E-07
14	FEV1 %pred	Pack-years	4.86E-24	0.0010	1.06E-07
15	FEV1 %pred	BMI	1.78E-16	0.0019	0.0003
16	FEV1 %pred	Age	7.75E-12	1.97E-05	0.0091
17	Exacerbation Frequency	6MWD	2.07E-11	0.0002	0.0009
18	Emphysema	Age	7.17E-10	9.36E-08	0.1643
19	FEV1 %pred	Emphysema	6.35E-07	0.8129	9.98E-06
20	Exacerbation Frequency	Age	9.39E-07	0.0013	0.0008
21	Emphysema	Emphysema Distribution	6.32E-06	0.0034	0.0050
22	Emphysema	BMI	9.07E-06	0.0807	0.0099
23	Airway Wall Area	Exacerbation Frequency	1.19E-05	0.6975	0.0002
24	Emphysema Distribution	Gas Trapping	1.90E-05	0.0265	0.0072
25	Gas Trapping	Pack-years	2.86E-05	0.0234	0.0002
26	Emphysema Distribution	6MWD	5.50E-05	0.1449	0.0185
27	Emphysema	Pack-years	0.0001	0.0457	0.0007
28	BMI	Age	0.0002	0.6948	0.9065
29	Emphysema	6MWD	0.0002	0.1266	0.0188
30	Gas Trapping	Exacerbation Frequency	0.0015	0.4982	0.0003
31	Gas Trapping	Airway Wall Area	0.0015	0.1315	0.2047
32	Emphysema Distribution	Age	0.0035	0.6610	0.0830
33	Airway Wall Area	Pack-years	0.0046	0.5833	0.9392
34	Emphysema Distribution	BMI	0.0068	0.0259	0.0494
35	FEV1 %pred	Emphysema Distribution	0.0166	0.5650	0.1842
36	BMI	Pack-years	0.0252	0.0117	0.0653
37	Airway Wall Area	BMI	0.0315	0.6317	0.1685
38	Exacerbation Frequency	BMI	0.0967	0.4106	0.7012
39	Exacerbation Frequency	Pack-years	0.1550	0.7941	0.2046
40	Gas Trapping	6MWD	0.2014	0.3027	0.6971
41	Airway Wall Area	Age	0.2467	0.5873	0.5646
42	Emphysema Distribution	Exacerbation Frequency	0.3621	0.0167	0.7624
43	Emphysema Distribution	Airway Wall Area	0.4888	0.2023	0.0958
44	Emphysema	Exacerbation Frequency	0.7714	0.1161	0.1067
45	Emphysema Distribution	Pack-years	0.8382	0.3054	0.3293

Table S3: p-values for all edges for non-Hispanic White (NHW) populations with 2 copies of COPD risk or COPD non-risk allele (FAM13A)

	Node 1	Node 2	Population PCOR	2 copies COPD risk allele PCOR	2 copies COPD non-risk allele PCOR	2 copies COPD risk allele perm p-value	2 copies COPD non-risk allele perm p-value
1	Emphysema	Gas Trapping	0.6541	0.6935	0.6667	0.1582	0.2672
2	FEV1 %pred	Gas Trapping	-0.3538	-0.4507	-0.3717	0.0372	0.0168
3	FEV1 %pred	Airway Wall Area	-0.3200	-0.3340	-0.3042	0.3576	0.3838
4	Gas Trapping	Age	0.2350	0.3146	0.2269	0.012	0.0116
5	FEV1 %pred	6MWD	-0.2285	0.2401	0.2429	0.9614	0.929
6	Gas Trapping	BMI	-0.1982	-0.2110	-0.1814	0.3906	0.4062
7	6MWD	BMI	-0.1857	-0.2343	-0.1919	0.2402	0.3108
8	Airway Wall Area	6MWD	0.1840	-0.1520	-0.1593	0.775	0.9706
9	Emphysema	Airway Wall Area	0.1796	-0.1311	-0.1558	0.4606	0.693
10	FEV1 %pred	Exacerbation Frequency	-0.1408	-0.1490	-0.1425	0.6618	0.9754
11	Age	Pack-years	-0.1405	0.1012	0.1392	0.3126	0.257
12	6MWD	Pack-years	-0.1316	-0.1617	-0.1036	0.1628	0.1662
13	6MWD	Age	-0.1151	-0.1326	-0.1422	0.732	0.8244
14	FEV1 %pred	Pack-years	-0.1118	-0.0895	-0.1480	0.1456	0.1306
15	FEV1 %pred	BMI	-0.0911	-0.0843	-0.1017	0.5994	0.7146
16	FEV1 %pred	Age	-0.0758	0.1158	0.0729	0.2186	0.3072
17	Exacerbation Frequency	6MWD	-0.0742	-0.1008	-0.0931	0.8598	0.8658
18	Emphysema	Age	-0.0683	-0.1446	-0.0389	0.0032	0.0036
19	FEV1 %pred	Emphysema	0.0552	-0.0064	-0.1232	6.00E-04	0.0006
20	Exacerbation Frequency	Age	-0.0543	-0.0876	-0.0937	0.755	0.9728
21	Emphysema	Emphysema Distribution	0.0500	0.0797	0.0785	0.9104	0.8252
22	Emphysema	BMI	0.0492	-0.0475	-0.0721	0.4476	0.4946
23	Airway Wall Area	Exacerbation Frequency	-0.0485	-0.0106	0.1056	0.1184	0.0138
24	Emphysema Distribution	Gas Trapping	-0.0474	-0.0604	-0.0751	0.6136	0.7288
25	Gas Trapping	Pack-years	-0.0464	0.0617	0.1055	0.308	0.2432
26	Emphysema Distribution	6MWD	0.0447	-0.0397	-0.0659	0.448	0.3946
27	Emphysema	Pack-years	0.0427	-0.0544	-0.0943	0.3566	0.2718
28	BMI	Age	-0.0419	0.0107	0.0033	0.8014	0.2414
29	Emphysema	6MWD	0.0408	-0.0416	-0.0657	0.4936	0.5466
30	Gas Trapping	Exacerbation Frequency	-0.0353	0.0184	0.1011	0.08	0.0578
31	Gas Trapping	Airway Wall Area	-0.0353	-0.0411	-0.0355	0.812	0.9528
32	Emphysema Distribution	Age	0.0324	-0.0119	-0.0485	0.3126	0.2676
33	Airway Wall Area	Pack-years	-0.0314	-0.0149	-0.0021	0.9902	0.132
34	Emphysema Distribution	BMI	-0.0300	-0.0606	-0.0550	0.8802	0.8502
35	FEV1 %pred	Emphysema Distribution	0.0266	-0.0157	-0.0372	0.5556	0.5866
36	BMI	Pack-years	0.0248	0.0686	0.0516	0.7038	0.6188
37	Airway Wall Area	BMI	-0.0238	0.0131	-0.0385	0.7652	0.2676
38	Exacerbation Frequency	BMI	-0.0184	0.0224	0.0107	0.8322	0.6014
39	Exacerbation Frequency	Pack-years	-0.0158	-0.0071	-0.0355	0.3584	0.4616
40	Gas Trapping	6MWD	0.0142	-0.0281	0.0109	0.3052	0.8064
41	Airway Wall Area	Age	0.0128	-0.0148	-0.0161	0.835	0.8718
42	Emphysema Distribution	Exacerbation Frequency	-0.0101	0.0651	-0.0085	0.1666	0.2312
43	Emphysema Distribution	Airway Wall Area	-0.0077	0.0347	0.0466	0.8472	0.6508
44	Emphysema	Exacerbation Frequency	0.0032	0.0428	-0.0451	0.1906	0.1876
45	Emphysema Distribution	Pack-years	-0.0023	0.0279	0.0273	0.987	0.9792

Table S3 (cont.): partial correlations and permutation-based p-values for all edges for non-Hispanic White (NHW) populations with 2 copies of COPD risk or COPD non-risk allele (FAM13A)
