# Supplementary material for: Analyzing networks of phenotypes in complex diseases: methodology and applications in COPD
Source: BMC Syst Biol. 2014 Jun 25;8:78. doi: 10.1186/1752-0509-8-78 (PMC4105829; doi:10.1186/1752-0509-8-78)
Supplement: Additional file 5 — Table S4. Raw p-values and partial correlations for all edges in COPDGene and ECLIPSE for all cases. [file 1752-0509-8-78-S5.pdf]

Node 1	Node 2	COPDGene P-value	COPDGene PCOR	ECLIPSE P-value	ECLIPSE PCOR
FEV1%PRED	Emphysema	5.40E-164	-0.4769	7.24E-79	-0.4337
FEV1%PRED	6MWD	2.40E-72	0.3257	5.65E-24	0.2415
FEV1%PRED	Airway Wall Area	1.67E-65	-0.3103	2.77E-15	-0.19
Emphysema	Airway Wall Area	3.07E-60	-0.2978	7.53E-19	-0.2128
Emphysema	Age	1.58E-46	0.2619	9.24E-10	0.1478
Emphysema	BMI	1.54E-41	-0.2474	0.0168	-0.058
6MWD	BMI	2.00E-22	-0.1799	1.60E-10	-0.1543
Age	Pack-years	2.23E-22	0.1797	9.61E-09	0.1386
Airway Wall Area	6MWD	4.97E-10	-0.1154	7.48E-05	-0.0959
FEV1%PRED	Exacerbation Frequency	3.75E-08	-0.1022	6.12E-06	-0.1094
6MWD	Age	3.83E-07	-0.0943	9.00E-10	-0.1479
Exacerbation Frequency	6MWD	2.17E-06	-0.088	2.11E-06	-0.1147
FEV1%PRED	BMI	3.51E-06	0.0862	3.14E-11	0.1602
Exacerbation Frequency	Age	5.41E-06	-0.0845	0.3529	-0.0226
6MWD	Pack-years	0.0006	-0.064	0.2428	-0.0284
Emphysema	Exacerbation Frequency	0.0015	0.059	0.3183	0.0242
Airway Wall Area	Exacerbation Frequency	0.002	0.0573	0.2788	-0.0263
FEV1%PRED	Age	0.0023	0.0567	3.39E-09	0.1428
BMI	Age	0.0223	0.0425	0.1398	-0.0358
Emphysema	Pack-years	0.0419	0.0378	0.1627	0.0339
Airway Wall Area	BMI	0.054	0.0359	1.04E-29	0.2697
Exacerbation Frequency	BMI	0.0635	0.0345	0.6702	-0.0103
BMI	Pack-years	0.0718	0.0335	0.0039	0.07
Emphysema	6MWD	0.3311	-0.0181	0.0012	-0.0784
Exacerbation Frequency	Pack-years	0.3314	-0.018	0.101	-0.0398
FEV1%PRED	Pack-years	0.3545	-0.0172	0.036	-0.0509
Airway Wall Area	Age	0.5199	-0.012	0.4327	0.019
Airway Wall Area	Pack-years	0.6522	0.0084	0.0144	0.0594

Table S4: p-values and partial correlations for all edges in COPDGene and ECLIPSE for all cases
