# Supplementary material for: Analyzing networks of phenotypes in complex diseases: methodology and applications in COPD
Source: BMC Syst Biol. 2014 Jun 25;8:78. doi: 10.1186/1752-0509-8-78 (PMC4105829; doi:10.1186/1752-0509-8-78)
Supplement: Additional file 6 — Table S5. Raw p-values and partial correlations for all edges in COPDGene and ECLIPSE for all white cases. [file 1752-0509-8-78-S6.pdf]

Node 1	Node 2	COPDGene P-value	COPDGene PCOR	ECLIPSE P-value	ECLIPSE PCOR
FEV1%PRED	Emphysema	3.60E-127	-0.4745	7.70E-77	-0.433
FEV1%PRED	6MWD	3.52E-75	0.3723	5.26E-23	0.239
FEV1%PRED	Airway Wall Area	2.77E-55	-0.321	9.99E-15	-0.1883
Emphysema	Airway Wall Area	1.62E-46	-0.2948	3.00E-18	-0.2115
Emphysema	Age	3.33E-32	0.2449	3.87E-09	0.1439
Emphysema	BMI	3.02E-31	-0.2412	0.0224	-0.056
6MWD	BMI	3.45E-22	-0.2019	3.17E-10	-0.1535
6MWD	Age	3.44E-14	-0.1586	7.95E-10	-0.1501
Age	Pack-years	1.53E-09	0.1267	4.41E-08	0.1338
6MWD	Pack-years	2.68E-09	-0.1248	0.3976	-0.0208
Exacerbation Frequency	Age	7.16E-09	-0.1214	0.2433	-0.0286
FEV1%PRED	BMI	4.22E-07	0.1062	2.02E-10	0.1552
Airway Wall Area	6MWD	1.06E-06	-0.1025	5.13E-05	-0.0992
Exacerbation Frequency	6MWD	1.36E-06	-0.1015	4.45E-07	-0.1235
FEV1%PRED	Age	3.24E-06	0.0978	1.03E-07	0.1301
FEV1%PRED	Exacerbation Frequency	4.77E-06	-0.0961	1.95E-05	-0.1046
Airway Wall Area	Exacerbation Frequency	0.0075	0.0562	0.2712	-0.027
Emphysema	Exacerbation Frequency	0.0084	0.0554	0.254	0.028
Emphysema	Pack-years	0.041	0.043	0.147	0.0356
BMI	Pack-years	0.1714	0.0288	0.0025	0.074
Exacerbation Frequency	Pack-years	0.1826	-0.0281	0.1256	-0.0376
Airway Wall Area	Age	0.3358	0.0203	0.4524	0.0184
BMI	Age	0.3373	0.0202	0.0944	-0.0411
Exacerbation Frequency	BMI	0.3889	0.0181	0.3743	-0.0218
Airway Wall Area	BMI	0.446	0.016	8.49E-30	0.2731
Airway Wall Area	Pack-years	0.544	0.0127	0.0202	0.057
Emphysema	6MWD	0.5888	0.0114	0.0011	-0.0802
FEV1%PRED	Pack-years	0.9407	-0.0016	0.01945	-0.0573

Table S5: p-values and partial correlations for all edges in COPDGene and ECLIPSE for all white cases
