# Supplementary material for: Analyzing networks of phenotypes in complex diseases: methodology and applications in COPD
Source: BMC Syst Biol. 2014 Jun 25;8:78. doi: 10.1186/1752-0509-8-78 (PMC4105829; doi:10.1186/1752-0509-8-78)
Supplement: Additional file 7 — Figure S2. Comparison of ECLIPSE and COPDGene networks on all cases and white cases only. Undirected edges denote partial correlation coefficients that were significant at p<0.001. [file 1752-0509-8-78-S7.pdf]

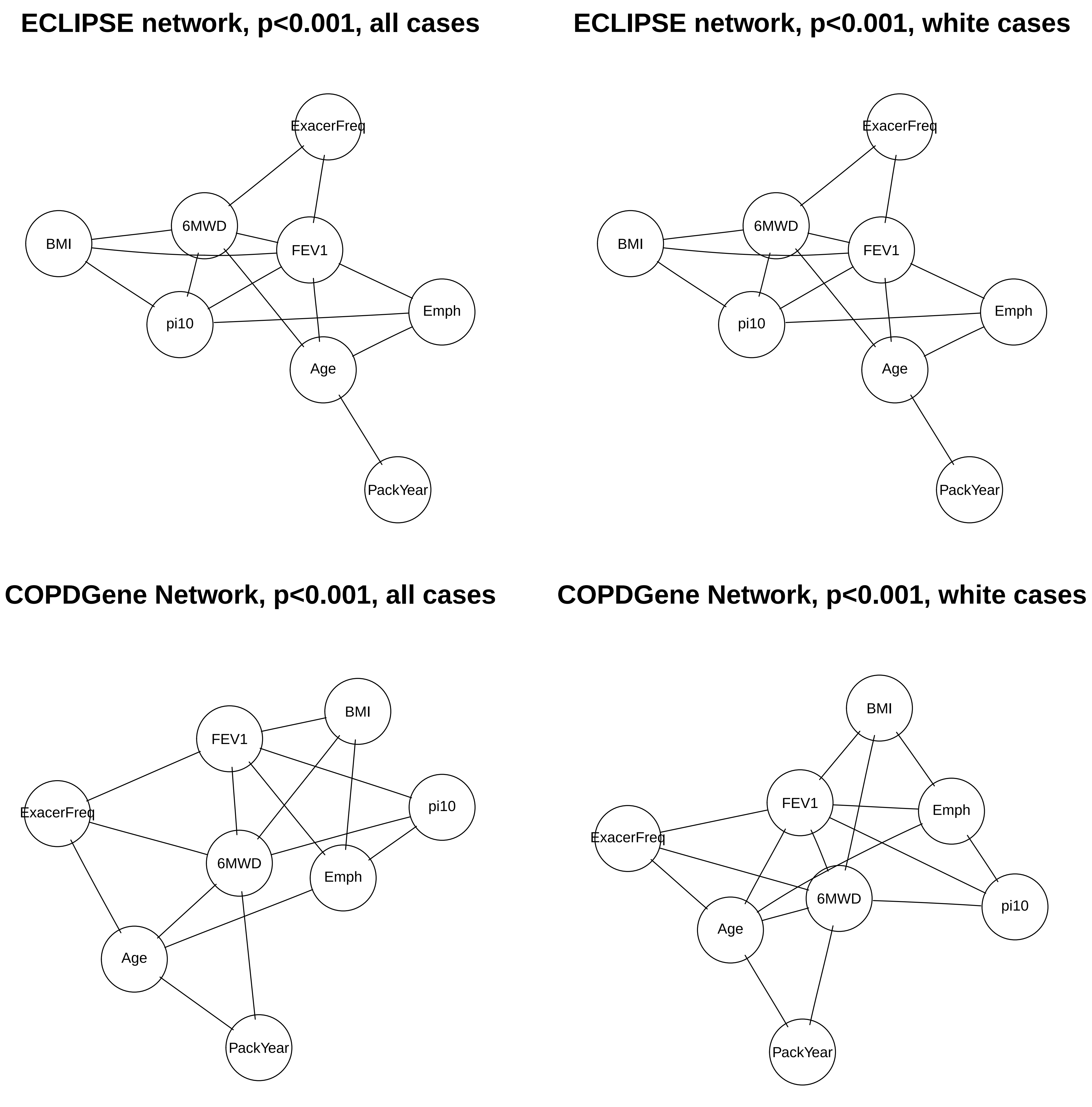
ECLIPSE network, p<0.001, all cases
ExacerFreq
BMI
6MWD
FEV1
pi10
Emph
Age
PackYear
ECLIPSE network, p<0.001, white cases
ExacerFreq
BMI
6MWD
FEV1
pi10
Emph
Age
PackYear
COPDGene Network, p<0.001, all cases
FEV1
BMI
ExacerFreq
pi10
6MWD
Emph
Age
PackYear
COPDGene Network, p<0.001, white cases
BMI
FEV1
Emph
ExacerFreq
6MWD
pi10
Age
PackYear
